# Susceptibility assessment of debris flow hazards from the perspective of watershed units grounded in the Random Forest (RF) model—a case study in the upper reaches of the Fujiang River

**DOI:** 10.1038/s41598-026-50681-1

**Published:** 2026-05-12

**Authors:** Weiwei Liu, Xiaoxian Lin, Xin Zhu

**Affiliations:** 1 College of Geography and Environment, Mianyang Teachers’ College, Mianyang, 621000 Sichuan China; 2 Daying Ecological Environment Monitoring Station, Suining, 629399 Sichuan China

**Keywords:** Random Forest model (RF), Geographic Information System (GIS), Susceptibility assessment, Debris flow, Upper reaches of the Fujiang River, Ecology, Ecology, Environmental sciences, Hydrology, Natural hazards

## Abstract

As a critical ecological barrier in the upper Yangtze River basin, the upper reaches of the Fujiang River face severe constraints on sustainable development as a consequence of frequent debris flow hazards. For this reason, this study utilized 685 watershed units as evaluation units in the region to accurately identify high-risk areas and core driving factors while establishing a scientific susceptibility assessment framework. Eleven hazard-inducing factors, including watershed area, average elevation, and watershed elevation difference, were integrated to construct a Random Forest (RF) model on the Python platform for debris flow susceptibility assessment and key factor diagnosis. We ultimately arrived at the following conclusions. (1) Watershed area and mean annual precipitation are the primary factors controlling debris flow development, with contribution rates of 0.109 each, followed by the Normalized Difference Vegetation Index (NDVI) and average elevation (both exceeding 0.104), while land use change has the lowest contribution rate (0.053); (2) The RF model demonstrates excellent evaluation accuracy, with an Area Under the Curve (AUC) value of 0.82 and an Accuracy (ACC) value of 0.82. The study area is classified into five susceptibility levels, namely, very low, low, moderate, high, and very high. Distributing in a zonal pattern along the slopes of the main stem of the Fujiang River and its tributaries, such as the Huoxi River and Baicao River, the very high susceptibility areas (579.04 km^2^) and high susceptibility areas (58.1 km^2^) are primarily concentrated in unstable valley regions in the northwestern part of Maoxian-Beichuan, the eastern part of Songpan County, and the southeastern part of Pingwu County; (3) Among the 144 debris flow hazard sites within the watersheds, 56.94% (82 sites) are located in very high susceptibility areas, and 27.78% (40 sites) are in high susceptibility areas, validating the reliability of the assessment results. This study innovatively employs watershed units as evaluation units, thereby overcoming the limitations of traditional grid and administrative units. It clarifies the spatial pattern and key driving mechanisms of debris flow susceptibility in the upper reaches of the Fujiang River, providing a scientific basis and technical support for precise disaster prevention and control, land-use planning, and ecological protection in mountainous regions of southwest China.

## Introduction

Debris flows are sudden and destructive geological hazards in mountainous regions. Aside from that, their susceptibility assessment has become a core component of disaster risk prevention and control as well as land-use planning^1,2^. As integrated carriers of hydrological and geomorphological processes, watershed units have emerged as the mainstream spatial scale for susceptibility modeling on account of their ability to accurately capture the complex interactions between topography, geology, and hydrology^3,4^. Notwithstanding the extensive optimization of the assessment framework and integrated multi-source data by global scholars, as well as the significant role this area plays as an ecological barrier, specialized studies have rarely reported to probe deep into key tributaries in the upper reaches of the Yangtze River, especially the upper reaches of the Fu River.

Research in this field has evolved from traditional statistical modeling methods, such as Frequency Ratio (FR) and Weight of Evidence (WoE)^5,6^, to advanced machine learning and multi-process coupling techniques^7^. Models like Random Forest (RF) and Decision Tree (DT) are highly regarded for their exceptional nonlinear fitting capabilities and stability in handling multi-factor interactions^8,9^. Recent studies published in Applied Sciences have further advanced this field by exploring innovative negative sample acquisition strategies for RF-based debris flow susceptibility mapping, demonstrating that approaches such as isolation forest (IF) methods are particularly well-adapted to watershed unit datasets^10,11^. Comparative analyses of machine learning algorithms in alpine-valley regions have also confirmed that RF outperforms support vector machine (SVM) models in terms of accuracy, with relative elevation difference emerging as the most prominent evaluation factor^12^. In China, relevant studies have immensely advanced indicator localization and model adaptability. Scholars have established multi-dimensional systems encompassing "topography-geology-hydrology-human activities", tailored to the characteristics of high-altitude regions^2,12^. Recent innovations include integrating Sentinel-1 radar data to overcome data acquisition challenges in cloudy mountainous areas^12^ and optimizing watershed units through hierarchical river network features^13^, jointly confirming the effectiveness of machine learning in complex terrains.

Despite the aforementioned breakthroughs, several critical issues persist: (1) Inconsistent watershed delineation standards hinder cross-regional comparisons; (2) A lack of specialized studies on the upper reaches of the Fujiang River has left its complex disaster-driving mechanisms poorly understood; (3) Existing models rarely throw light upon the synergistic effects among environmental driving factors in a quantitative manner. Located in the tectonically active transition zone between the Qinghai-Tibet Plateau and the Sichuan Basin, the upper reaches of the Fujiang River face frequent disasters and intensifying human activities^14,15^. In an effort to mitigate these research gaps, this present study not only adopts watershed units as the fundamental evaluation units, but also employs the RF model to assess debris flow susceptibility. By integrating 11 multi-source factors, this research is predominantly intended to identify key regional drivers and provide scientific support for disaster prevention and mitigation as well as ecological protection in the upper Yangtze River basin.

## Overview of the study area

**Fig. 1:**
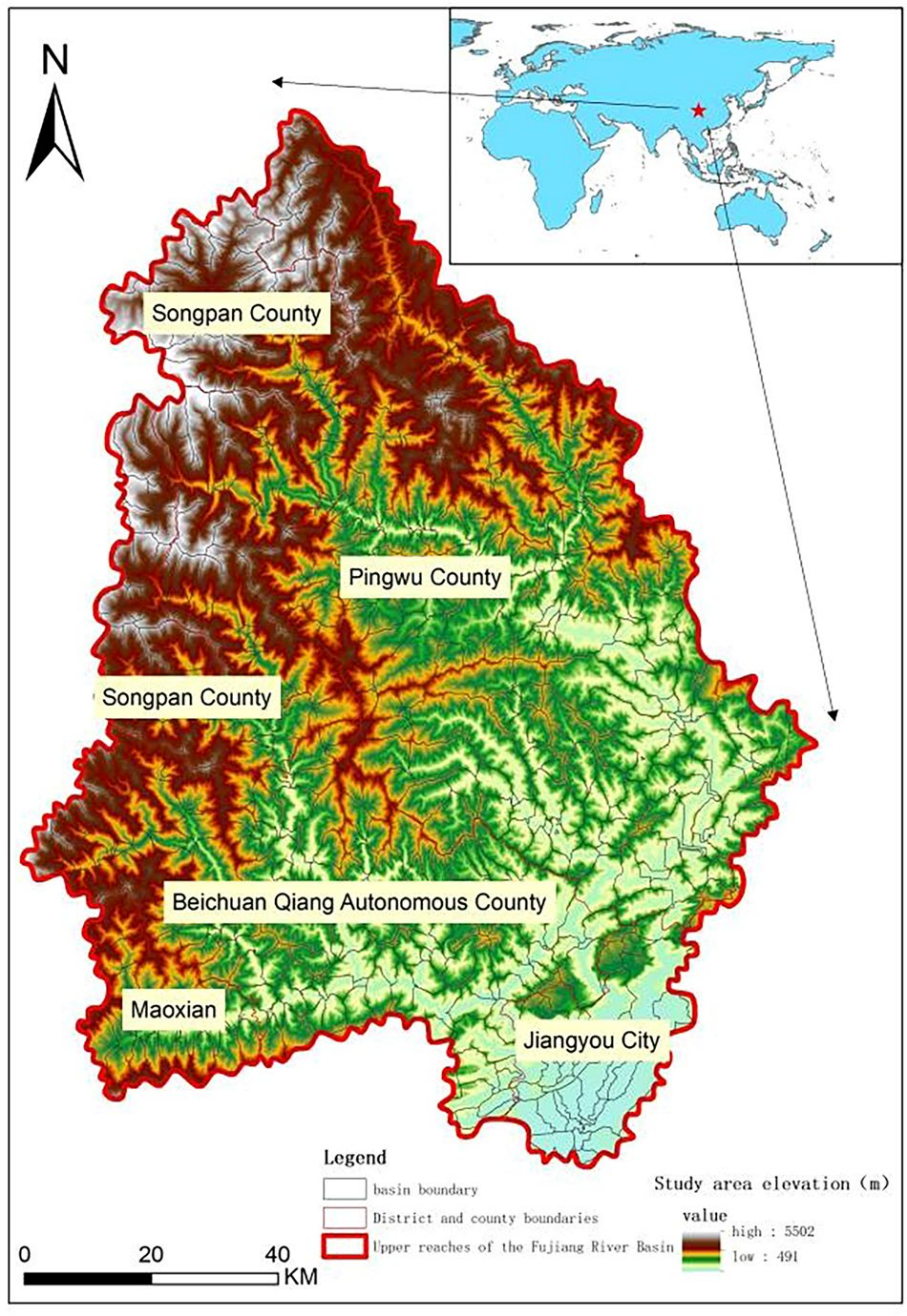
Schematic diagram of the division of debris flow basin units in the upper reaches of the Fujiang River.

Located in northwestern Sichuan Province, China, the upper reaches of the Fujiang River serve as the source and core flow region of the Fujiang River, which is a secondary tributary of the Yangtze River. This area is not only geographically unique but also historically significant as a vital corridor connecting central China with the southwest, boasting rich natural landscapes and cultural heritage. As demonstrated in Fig. 1, the upper reaches of the Fujiang River (103°45′–105°15′ E, 31°40′–33°10′ N) refer to the watershed region from the source of the Fujiang River to the control section of the Wudu Town hydrological station in Jiangyou City. The source is located at Xuebaoding, the main peak of the Minshan Mountains, with the main stem flowing southward and major tributaries including the Huoxi River, Huya River, and Pingtong River, covering a total area of approximately 19,000 km^2^. The Fujiang River is a primary tributary on the right bank of the upper Yangtze River, originating from Sanchazi at the northern foot of Xuebaoding, the main peak of the Minshan Mountains, in Songpan County, Aba Tibetan and Qiang Autonomous Prefecture, Sichuan Province. The regional topography exhibits pronounced step-like and transitional characteristics, transitioning from plateaus and high mountains in the northwest to basins in the southeast, which displays extreme elevation differences. The highest point is Xuebaoding in Songpan County, at 5588 m above sea level, while the lowest point is in southern Jiangyou City, at approximately 500–600 m above sea level^14,16,17^.

Major fault zones in the upper reaches of the Fujiang River include the Longmenshan Fault Zone, Huya Fault Zone, and Minjiang Fault Zone^18^. Through data provided by the Sichuan Provincial Department of Natural Resources, remote sensing interpretation, and field surveys, a total of 192 debris flow hazard sites have been identified in the upper reaches of the Fujiang River, predominantly distributed across 34 townships in five counties within the two cities of Mianyang and Aba Tibetan and Qiang Autonomous Prefecture^19,20^.

## Data and research methods

### Data sources

The primary data include debris flow hazard sites, elevation (DEM), and other datasets, with specific sources detailed in Table 1. DEM remote sensing imagery of the Fujiang River basin was processed through geometric correction and delineated using watershed boundaries to define the upper reaches of the Fujiang River.

**Table 1:** Data sources.

Data type	Parameters	Source
Debris flow hazard sites	1:500,000	Sichuan Provincial Department of Natural Resources
NDVI data	30 m resolution	MOD13A1.006 Terra Vegetation Indices 16-Day Global 500 m dataset on GEE
Land cover data (2017–2024)	30 m resolution	Wuhan University Land Cover Dataset on CLCD
Meteorological data	30 m resolution	National Earth System Science Data Center (http://www.geodata.cn/)
DEM data	30 m resolution	ASTER GDEM30M Digital Elevation Data
Vegetation type data	1: 1,000,000 scale	Resource and Environment Science and Data Center, CAS (https://www.resdc.cn/)
Hydrological data		
Land use data (2000–2024)	30 m resolution	Professors Yang Jie and Huang Xin from Wuhan University published (10.5281/zenodo.18180184)

### Selection of evaluation units and indicators

#### Selection of evaluation units

As different units yield varying results, selecting appropriate evaluation units is crucial for debris flow susceptibility assessment. For the time being, grid units, watershed units, and administrative units are extensively utilized for susceptibility assessments of geological hazards like debris flows and landslides. Notwithstanding their conspicuous advantages like rapid subdivision and computation on the basis of GIS, grid units fail to link with the geological environment of debris flow regions, resulting in evaluations lacking practical attributes^18^. In contrast, watershed units effectively integrate the geological environment of debris flow regions^21^, which not only takes into consideration topographic and material source conditions for debris flow formation and development, but also facilitates the extraction of susceptibility assessment indicators and factor values to yield reliable results. Administrative units conduct evaluations rooted in entire administrative regions, which is advantageous for government departments at all levels to formulate disaster prevention and mitigation policies, and thereby conduct disaster relief efforts^22^. Nonetheless, their results not only fail to correlate with actual topographic and geomorphic features, but also cannot truly reflect the relationship between debris flows and assessment factors. To this end, this study selects watershed units as the evaluation units for debris flow susceptibility in the upper reaches of the Fujiang River. Using ArcGIS hydrological analysis functions^23^, we compared river networks extracted under different thresholds (1000, 2000, 3000) with the actual river network distribution in the study area. We arrived at a conclusion that a threshold of 1000 yielded a river network and surrounding watersheds largely consistent with the actual distribution. For this reason, a threshold of 1000 was used to extract the river network, ultimately dividing the upper reaches of the Fujiang River into 685 watershed units with an average area of 18 km^2^. Figure 1 depicts the distribution of debris flow watersheds in the upper reaches of the Fujiang River.

#### Selection of evaluation indicators

The development of debris flows requires at least three conditions: ① steep slopes, ② a large amount of loose materials, and ③ an adequate water source. Steep slopes are one of the crucial conditions for debris flow development, as they provide sufficient potential energy, enabling the rapid flow of debris flows under the influence of gravity. Debris flows are typically formed by the mixture of water and mud in slopes or gullies containing a large amount of loose materials (such as soil, sand, rock debris, etc.). As substantial rainfall, snowmelt, mountain meltwater, or groundwater outbursts can provide sufficient water sources, water functions as a significant factor in debris flow development, thereby allowing loose materials to mix with water and form debris flows. As a result, the selection of evaluation factors for debris flow susceptibility should take into account the scientificity, practicality, and correctness of the factors. Grounded in the relevant explanations in Chapter 6 *Survey Content of Disaster-Pregnant Geological Conditions* of the Technical Requirements for Geological Survey (1:50,000) by the China Geological Survey Bureau^24^, as well as relevant research findings on the causes of debris flow disasters in China, and considering the actual situation of the study area, factors such as topography and geomorphology, meteorology and hydrology, land use change, and vegetation coverage were selected as evaluation factors for debris flow susceptibility in the upper reaches of the Fujiang River. Taking into account the availability of each evaluation factor and combining with the debris flow basin units in the study area, 11 evaluation factors were ultimately selected to construct an evaluation system for debris flow susceptibility in the upper reaches of the Fujiang River for susceptibility assessment.

#### Quantitative grading standards for evaluation factors

As a result of the different evaluation criteria for each indicator, involving qualitative and quantitative, absolute and relative quantities, and differences in units of measurement, as well as considering the consistency and rationality of subsequent overlay analysis of layers, reference was made to the Technical Requirements for Geological Survey (1:50,000) by the China Geological Survey Bureau^25^ and relevant research results by Zhu Xiaolong et al. Each indicator’s corresponding disaster risk level was classified, ranging from extremely low to extremely high, unified as Grades I–V, as revealed in Table 2.

**Table 2:** Quantitative grading table for factor indicators.

Evaluation factor indicator	Evaluation indicator grading (risk level)				
	Extremely low (I)	Low (II)	Medium (III)	High (IV)	Extremely high (V)
Basin area/km^2^	0.006–7.387	7.38–17.63	17.63–30.45	30.45–51.99	51.99–134.34
Average elevation/m	493–1200.07	1200.07–1816.88	1816.88–2523.955	2523.95–3291.20	3291.20–4329.25
Basin elevation difference/m	0–689	689–1253	1253–1693	1693–2239	2239–3388
Average slope/(°)	0.53–10.27	10.27–21.43	21.43–26.39	26.39–30.11	30.11–45.70
Average plan curvature	− 0.79 to − 0.43	− 0.43 to 0.09	− 0.09 to 0.006	0.006–0.029	0.029–0.413
Average profile curvature	− 0.20 to 0.002	0.002–0.015	0.015–0.056	0.056–0.252	0.252–1.523
Melton ratio	0–0.214	0.124–0.567	0.567–0.846	0.846–1.368	1.368–3.178
NDVI index	0.017–0.226	0.226–0.332	0.332–0.429	0.429–0.528	0.528–0.681
Land use change	WL to WL, BL to BL	FL to FL, SL to SL, FL to SL	FL or SL to GL, GL to GL	FL or SL or GL to CL, CL to GL	FL or SL or GL or CL to BA or BL
Average annual precipitation/mm	679.70–748.66	748.66–778.93	778.93–809.21	809.21–849.58	849.58–894.15
Average annual temperature/℃	− 4.61 to 2.13	2.13–6.18	6.18–9.64	9.64–13.02	13.02–16.90

## Results

The Random Forest (RF) model is an ensemble learning method employed to solve classification and regression problems^26–29^. It consists of multiple decision trees, each constructed rooted in a randomly selected subset of features and randomly selected training samples.

### Analysis of the area proportion and spatial distribution of risk levels for evaluation indicators

**Fig. 2:**
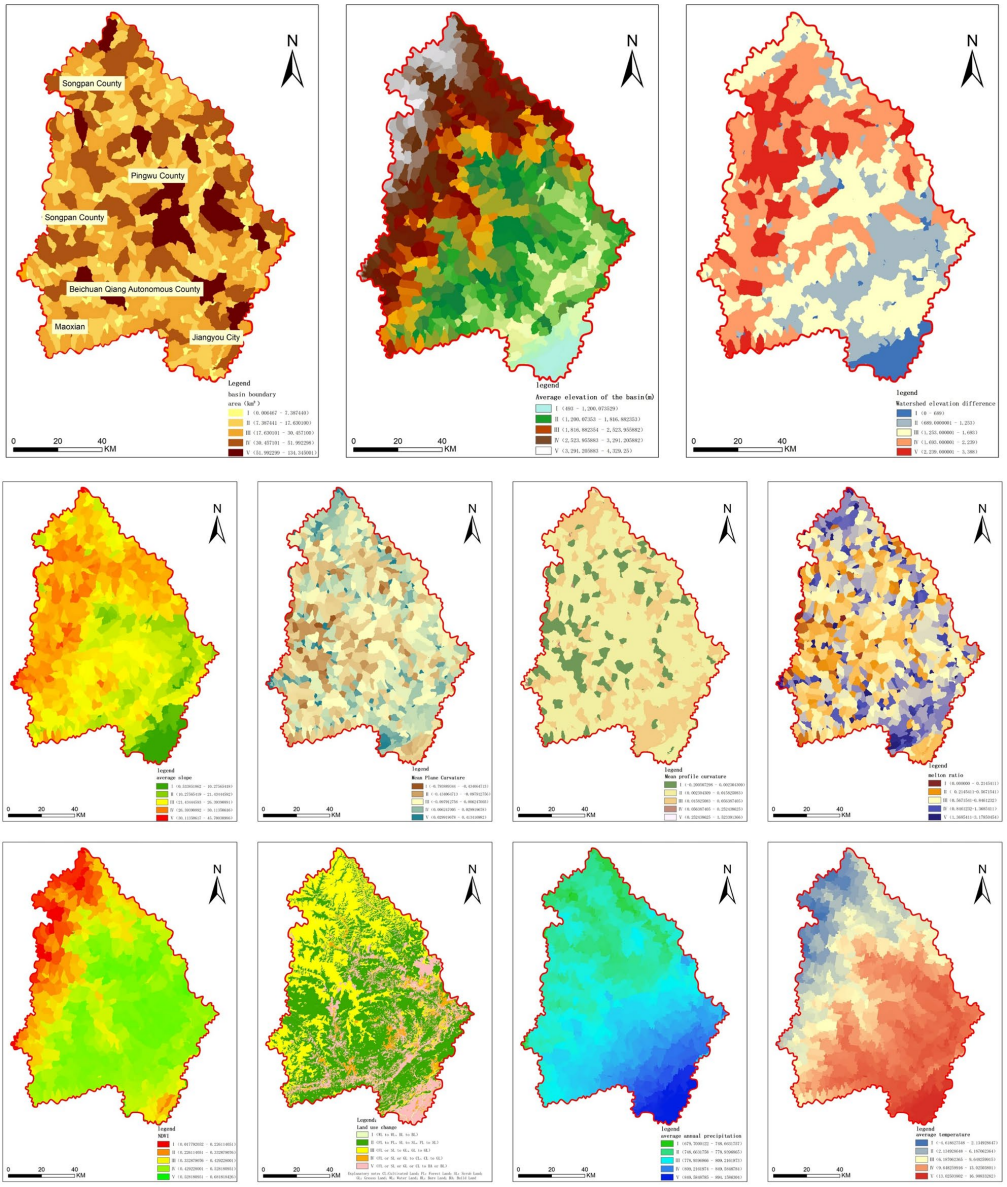
Grading map of debris flow basin factors.

As illustrated in Fig. 2, the spatial distribution of risk levels for each indicator was classified and visualized in accordance with the aforementioned grading standards. By utilizing spatial statistical tools, the area and proportion of regions with different risk levels for each evaluation factor were summarized, with the results revealed in Table 3. As evidently demonstrated by the results, debris flow disasters in the upper reaches of the Fujiang River are primarily influenced by basin area and average annual precipitation, which are the largest controlling factors. Secondary influencing factors mainly include NDVI, average elevation, and average elevation difference, while average annual temperature and land use change have the least impact. Debris flow disaster points are mainly concentrated in areas with slopes ranging from 26.39° to 30.11° and elevations between 1,200.07 and 1816.88 m, which are medium-elevation regions. A large basin elevation difference provides suitable topographic conditions for debris flow disasters. Topographic profile within the basin exhibits strong incision, with towering peaks and deep gullies, resulting in strong hydrodynamic forces that easily carry material sources, thereby triggering debris flow disasters. The size of the basin area and average annual precipitation directly affect the amount of rainfall accumulated within the basin. The more rainfall accumulates in a debris flow basin, the more likely it is to carry enormous slope debris and sediment, further increasing the likelihood of debris flow disasters.

**Table 3:** Area and proportion of risk levels for each evaluation factor.

Evaluation factor indicator	Evaluation indicator grading (risk level)									
	Extremely low (I)		Low (II)		Medium (III)		High (IV)		Extremely high (V)	
	Area /km^2^	Proportion %	Area /km^2^	Proportion %	Area /km^2^	Proportion %	Area /km^2^	Proportion %	Area /km^2^	Proportion %
Basin area/km^2^	346.71	2.85	3049.73	25.07	3864.37	31.77	2859.53	23.51	2044.38	16.80
Average elevation/m	1401.42	11.52	4183.14	34.39	2565.78	21.09	2387.11	19.62	1627.27	13.38
Basin elevation difference/m	417.59	3.43	2063.73	16.96	4225.97	34.74	3873.65	31.85	1583.78	13.02
Average slope/(°)	333.33	2.74	1499.31	12.33	3399.64	27.95	4021.61	33.06	2910.83	23.92
Average plan curvature	1190.71	9.79	4292.07	35.28	4591.36	37.75	1980.15	16.28	110.43	0.90
Average profile curvature	1460.68	12.01	7491.22	61.57	3187.87	26.21	24.08	0.2	0.87	0.01
Melton ratio	365.16	3	2830.47	23.27	3246.75	26.69	4342.54	35.70	1379.80	11.34
NDVI Index	3680.38	30.25	2958.98	24.32	2041.04	16.78	2201.28	18.1	1283.04	10.55
Land use change/km^2^	329.5821	2.71	5644.799654	46.40	590.0787	28.71	3097.65	4.85	2107.2861	17.32
Average annual precipitation/mm	1803.05	14.82	2623.54	21.57	2908.72	23.91	3315.46	27.25	1513.95	12.45
Average annual temperature/℃	1301.25	10.7	2517.57	20.7	2320.43	19.08	2672.62	21.97	3352.85	27.55

### Analysis of factor contribution rates

In this study, data from 192 debris flow disasters in the upper reaches of the Fujiang River were used as research samples. Using ArcGIS10.7, DEM data for the upper reaches of the Fujiang River basin was divided into 685 basin units. A marker column attribute was added to each basin unit, with basin units where debris flows had occurred marked as "1" and those where debris flows had never occurred marked as "0". Consequently, the problem of debris flow susceptibility evaluation was transformed into a binary classification problem^30^. As the results illustrate, there were 33 basin units where debris flows had occurred and 628 basin units where debris flows had never occurred in the upper reaches of the Fujiang River. Initially, through literature review, summarizing previous experiences, and continuous testing, the basic dataset was divided into an 80% training set and a 20% test set in an 8:2 ratio for training and testing the debris flow susceptibility evaluation model in the upper reaches of the Fujiang River.

The Random Forest model was constructed by adopting the Scikit-learn framework built into the Python language. After hyperparameter tuning, the number of decision trees in the Random Forest model was set to 100, the splitting metric was “gini”, the maximum tree depth was “None”, the maximum number of features was “auto”, and the number of parallel jobs was “None”.

**Fig. 3:**
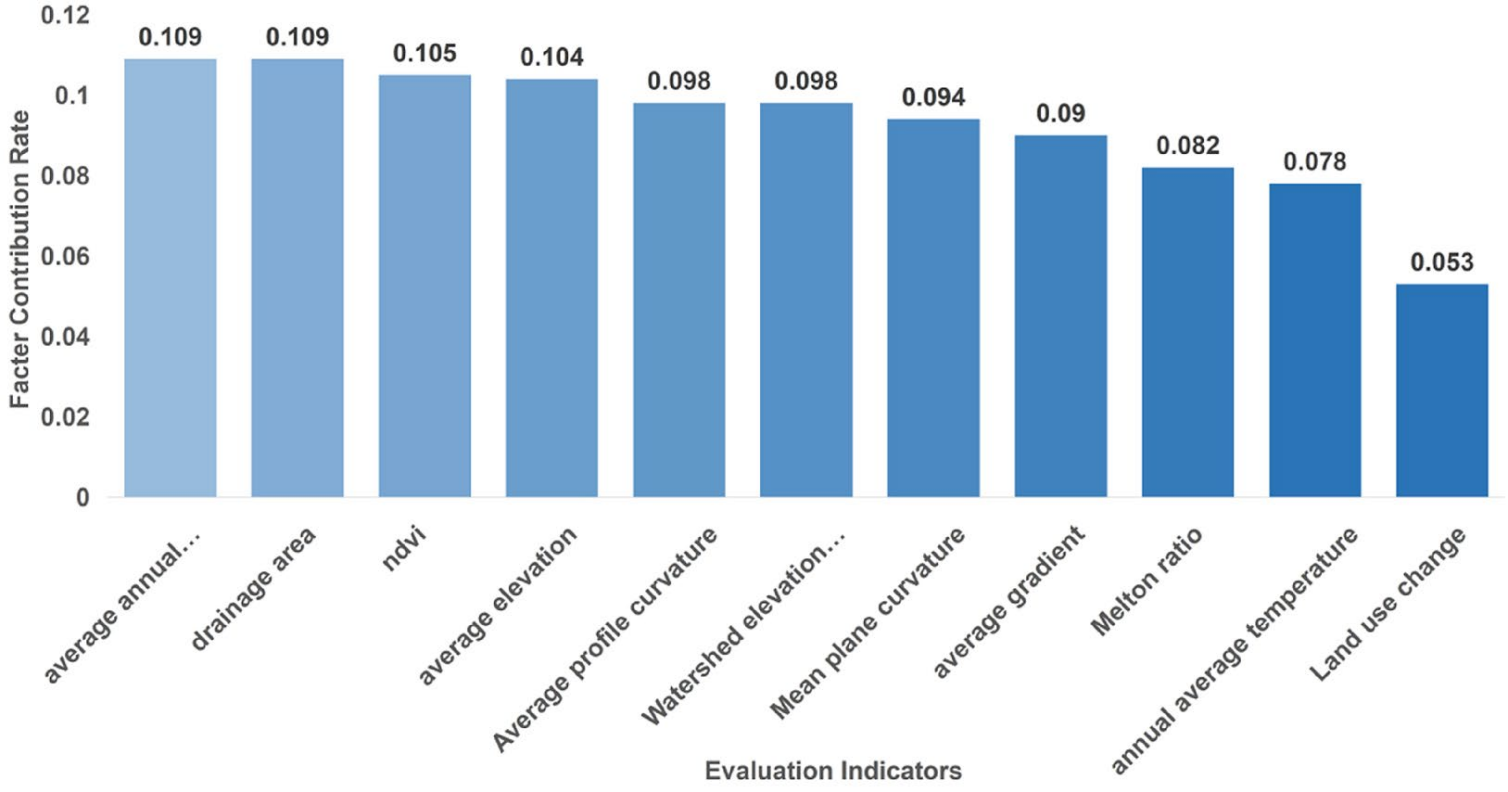
Factor contribution rates.

The factor contribution rates of the evaluation indicators included in the Random Forest model after hyperparameter tuning are depicted in Fig. 3. As suggested by an all-round observation, the factor contribution rates of basin area and average annual precipitation are the same, both at 0.109, making them the factors with the highest contribution rates among the evaluation indicators for inducing debris flow outbreaks. This also confirms that basin area and average annual precipitation have significant impacts on debris flow outbreaks in the basin. Land use change has the lowest contribution rate, at 0.053, in inducing debris flow outbreaks.

The contribution rates of the two factors, watershed area and annual average precipitation, display the same and relatively high values. This phenomenon suggests that watershed area and annual average precipitation may have significant impacts on the analysis of debris flow susceptibility. Aside from that, larger watershed areas and higher annual average precipitation may be associated with the occurrence of debris flows to a certain extent. NDVI is an indicator for measuring the condition of surface vegetation. A higher NDVI value indicates more luxuriant vegetation cover. As vegetation can stabilize soil and reduce the likelihood of soil erosion and debris flows, higher NDVI values may be correlated with the mitigation or prevention of debris flows. Both the average elevation and the elevation difference within the watershed are terrain-relevant. Steep terrain and large elevation changes may give rise to soil erosion and the occurrence of debris flows. Larger profile curvature, plan curvature, and slope potentially heighten the risk of soil erosion and debris flows. A higher Melton ratio may suggest a certain correlation between steep terrain and debris flow susceptibility. The contribution rates of the two factors, annual average temperature and land use change, are relatively low, suggesting that they may not have obvious direct impacts on the occurrence of debris flows. In summary, on the basis of the given factor contribution rates, watershed area, annual average precipitation, NDVI, average elevation, watershed elevation difference, average profile curvature, average plan curvature, average slope, and Melton ratio may be important factors influencing debris flow susceptibility. Simultaneously, other factors such as annual average temperature and land use change, with relatively low contribution rates, may conduct a trivial role in the analysis of debris flow susceptibility.

### Debris flow susceptibility analysis

**Fig. 4:**
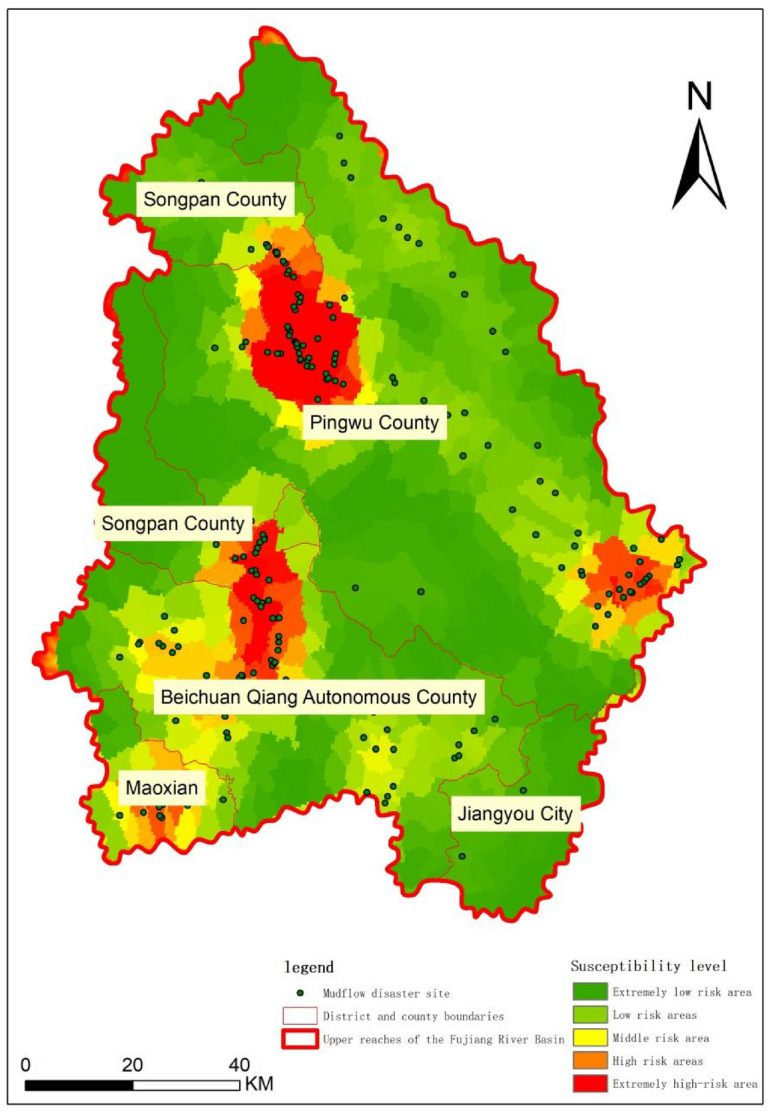
Susceptibility assessment map of debris flow disasters in the upper reaches of the Fujiang River.

Afterwards, a susceptibility assessment of debris flows in the upper reaches of the Fujiang River was conducted grounded in the Random Forest model. After the model ran, susceptibility index values were obtained for 685 watershed units in the upper reaches of the Fujiang River. By importing these susceptibility values into ArcGIS 10.7 software, the debris flow susceptibility values in the upper reaches of the Fujiang River were classified into five different levels according to the susceptibility index of the watershed units, namely, extremely low susceptibility area, low susceptibility area, moderate susceptibility area, high susceptibility area, and extremely high susceptibility area. A susceptibility assessment map of debris flow disasters in the upper reaches of the Fujiang River was subsequently generated (Fig. 4).

As evidenced in the figure, we can draw several conclusions below: (1) The extremely high and high susceptibility areas for debris flows in the upper reaches of the Fujiang River are mainly distributed in the northwestern part from Mao County to Beichuan, the eastern part of Songpan County, and the southeastern part of Pingwu County. They are mainly concentrated in extremely unstable valley areas, mostly distributed along the slopes on both sides of the main stream of the Fujiang River (from Songpan to Pingwu section), Huoxi River, Baicao River, and other rivers. (2) The moderate susceptibility areas for debris flows in the upper reaches of the Fujiang River are mainly distributed around the high susceptibility areas. This region is mainly mountainous. Since other geological disasters are also distributed in this area, they further provide material sources for debris flow disasters. Under specific conditions such as heavy rainfall scouring, debris flow disasters are likely to occur. (3) The extremely low and low susceptibility areas for debris flows in the upper reaches of the Fujiang River are mainly distributed in the central, northern, and eastern parts of the upper reaches of the Fujiang River, as well as in most areas of Jiangyou City. The terrain in this region is relatively gentle, with bedrock exposed on the surface, lacking the conditions for debris flow development.

With regard to the number of watersheds (Table 4), considering both the number and area of watersheds, the susceptibility zoning in the upper reaches of the Fujiang River is highly consistent. The extremely low susceptibility area occupies an absolutely dominant position, with both the number of watersheds (635) and the area (11,282.63 km^2^) accounting for 92.75% of the total. The extremely high susceptibility area comes next, with both indicators (31 in number and 579.04 km^2^ in area) accounting for 4.76%. The combined proportion of the other three susceptibility areas (moderate, low, and high) is less than 2.5%.

**Table 4:** Statistics on debris flow susceptibility in the watersheds of the upper reaches of the Fujiang River.

Risk zone	Number of watersheds/units	Watershed area/km^2^	Area proportion/%
I (extremely low)	635	11,282.63	92.75
II (low)	4	74.05	0.61
III (moderate)	10	170.89	1.40
IV (high)	3	58.10	0.48
V (extremely high)	31	579.04	4.76
Total	685	12,164.72	100

As suggested by comprehensive statistics on the 144 debris flow watersheds in the upper reaches of the Fujiang River and their susceptibility level zoning (Table 5).

**Table 5:** Statistics on debris flow susceptibility in the upper reaches of the Fujiang River.

County-level administrative/prone level	Extremely low susceptibility	Low susceptibility	Moderate susceptibility area	High susceptibility area	Extremely high susceptibility area
Jiangyou City	1	0	0	1	0
Pingwu County	1	4	3	11	41
Beichuan County	0	2	7	14	21
Maoxian County	0	0	0	4	8
Songpan County	2	0	2	10	12

### Model accuracy evaluation

**Fig. 5:**
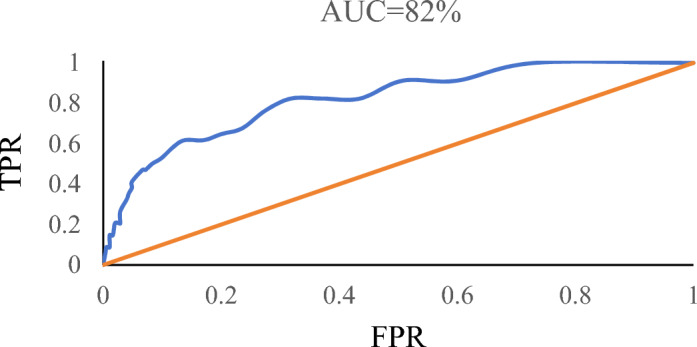
ROC curve.

The Receiver Operating Characteristic (ROC) curve (Fig. 5) is a tool employed to assess the performance of classification models. In this study, with the upper reaches of the Fujiang River as the target area, an innovative approach was adopted by using watershed units as the basic evaluation units. Rooted in a catchment threshold of 1000, the study area was divided into 685 watersheds, among which extremely low/low susceptibility areas accounted for 93.36%, and extremely high/high susceptibility areas accounted for 5.24%. By integrating 11 disaster-causing factors from four major categories, namely topography and geomorphology, meteorology and hydrology, vegetation coverage, and geology and soil, a Random Forest (RF) model was constructed on the Python platform to evaluate debris flow susceptibility^31–33^. Afterwards, the ROC curve and the Area Under the Curve (AUC) were employed to evaluate the model’s performance^34^. The ROC curve measures classification effectiveness by plotting the relationship between the True Positive Rate (TPR) and the False Positive Rate (FPR) at different thresholds, with a curve closer to the top-left corner indicating better performance. Verified by 144 historical debris flow disaster points, the model achieved an AUC value of 0.944 and an Accuracy (ACC) value of 0.82. On top of that, 56.94% of the disaster points were concentrated in extremely high susceptibility areas, and 27.78% were located in high susceptibility areas, thereby confirming the reliability and favorable applicability of the evaluation results, which can provide a scientific basis for precise prevention and control of debris flows in mountainous regions of Southwest China.

## Discussion

### Optimization and rationality comparison of evaluation units

The rational selection of evaluation units is crucial for the elevation of assessment accuracy. In general, traditional grid units are not only disadvantageous for the integrity of the geological environment, but are also challenging to reflect the continuous process of debris flow, which involves "catchment-sediment production–transportation". As a result of their artificial boundaries, administrative units fail to align with the natural continuity of the terrain, often leading to distorted results^14,17^. In this study, river networks were extracted using a threshold of 1000, and 685 watershed units (with an average area of 18 km^2^) were delineated through the hydrological analysis function of ArcGIS. This approach not only preserves the coupling relationships among terrain, hydrology, and material sources but also matches the patterns of disaster development, effectively overcoming the limitations of traditional units. In contrast to the study by Wang Shige et al.^12^ in the Dadu River basin^35^, this study incorporates additional factors such as average planar curvature, Melton ratio, and annual average temperature, providing a more comprehensive coverage of the material sources, hydrodynamic forces, and topographic base conditions for debris flow formation, aligning with the complex mountainous characteristics of the upper reaches of the Fujiang River. In comparison with the study by Zhu Xin et al.^36^, which used a catchment threshold of 3000 to divide 2948 fragmented units (with an average area of 4 km^2^), the units in this study are of moderate size, reducing data processing redundancy costs while avoiding the dissection of natural watershed coupling relationships. The susceptibility zoning is clear (extremely low/low susceptibility areas account for 93.36%, and extremely high/high susceptibility areas account for 5.24%), facilitating the precise identification of core risk areas and the formulation of prevention and control measures.

### Advantages of model selection and identification of core disaster-causing factors

The choice of model and factor combination can exert direct influences on the objectivity of the evaluation. Traditional methods such as the Analytic Hierarchy Process (AHP) and the Frequency Ratio (FR) method rely on subjective weighting or single statistical rules, thereby rendering it challenging to quantify the nonlinear interactions among multiple factors^37,38^. Some machine learning models suffer from inadequate factor screening specificity; for example, Wilford et al.'s model focused solely on topographic factors^39^, and Rowbotham’s logistic regression model lacked vegetation and soil parameters^40^. The RF model selected in this study possesses strong feature screening and nonlinear fitting capabilities, enabling it to automatically identify core driving factors and avoid human interference. This advantage aligns with recent findings in Applied Sciences, where machine learning-based feature importance analysis has been increasingly integrated with hyperparameter optimization techniques such as Bayesian Optimization to enhance both predictive performance and model explainability in geohazard assessments^41^. The integration of feature importance analysis with susceptibility modeling, as demonstrated in this study, provides clearer insight into the model’s decision-making process and enables more reliable engineering interpretation^41^. As the above results demonstrate, watershed area and annual average precipitation are the primary driving factors (both with a contribution rate of 0.109), aligning with the regional characteristics of "high mountains, deep valleys, and concentrated precipitation". Larger watershed areas provide space for the accumulation of loose materials and water collection, while concentrated precipitation supplies the hydrodynamic force for debris flow initiation. The Normalized Difference Vegetation Index (NDVI) and average elevation are secondary key factors (both with a contribution rate exceeding 0.104), reflecting the regulation of soil stability by vegetation and the disaster-causing characteristics of steep terrain and abundant material sources in high-altitude areas. land use change has the lowest contribution rate (0.053), which can be attributable to the fact that the soil in the study area is predominantly purple soil and skeletal soil with weak erosion resistance, and the impact of land use change differences on disasters is relatively uniform. This result accurately reveals the region-specific driving mechanisms, successfully addressing gaps in previous studies^18,42^. The contribution patterns observed in our study—where topographic conditions serve as prerequisites while precipitation and vegetation factors modulate disaster occurrence—are consistent with recent machine learning-based susceptibility assessments in alpine-valley regions documented in Applied Sciences and related publications. These studies have similarly emphasized that topographic conditions are fundamental prerequisites for debris flow development, while factors such as precipitation, vegetation cover, and anthropogenic influence play critical roles in determining the spatial distribution of high-susceptibility zones.

### Spatial pattern characteristics of susceptibility and causal analysis

The spatial pattern of debris flow susceptibility in the upper reaches of the Fujiang River is tightly correlated with the geological environment and human activities. Extremely high susceptibility areas (579.04 km^2^) and high susceptibility areas (58.1 km^2^) are concentrated in unstable valley regions in the northwestern part of Maoxian-Beichuan, the eastern part of Songpan County, and the southeastern part of Pingwu County, exhibiting a zonal distribution along the slopes on both sides of the main stream and tributaries of the Fujiang River^43^. Characterized by active geological structures and fragmented rock masses, this region lies within the influence range of the Longmenshan and Huya fault zones^13,44^. Coupled with significant topographic relief (up to 3388 m), average slopes ranging from 26.39° to 45.70°, and annual average precipitation between 849.58 and 894.15 mm, multiple factors contribute to the high incidence of disasters. Moderate susceptibility areas are distributed in the high mountains surrounding the high susceptibility areas, where material sources and hydrodynamic conditions are weaker, and disasters are likely to occur only under extreme rainfall conditions. Extremely low/low susceptibility areas account for 93.36% of the total area and are concentrated in the gentle regions of Jiangyou City and the central and northern parts of the basin, where the terrain is flat and bedrock is exposed, lacking the conditions for debris flow development. This pattern is consistent with the geomorphic characteristics and fault activity response laws revealed by Chen Hao et al.^20^, further confirming the rationality of the evaluation results.

### Comparative advantages of Random Forest (RF) versus XGBoost and SVM models

In the selection of machine learning models for debris flow susceptibility assessment^45^, the Random Forest (RF) model demonstrates comprehensive advantages better adapted to the watershed unit scale compared with XGBoost and Support Vector Machine (SVM). From the perspective of model mechanism, SVM relies on kernel functions to map high-dimensional spaces for classification, exhibiting weak fitting performance in scenarios with imbalanced samples and multi-factor coupling. When applied to the high-dimensional dataset of this study (685 watershed units and 11 evaluation factors), SVM tends to suffer from overfitting and reduced generalization ability. As a gradient boosting tree model, XGBoost enhances residual learning through serial iteration and performs well in small-sample fine prediction. However, it insufficiently captures the nonlinear factor interactions at the watershed scale, is sensitive to hyperparameters during training, and incurs higher tuning costs.The RF model in this study is centered on parallel decision tree ensembles and random feature sampling, featuring strong nonlinear fitting capability and quantifiable factor contribution analysis. It shows clear superiority in evaluation accuracy, stability and interpretability: the model achieves an AUC value of 0.82 and an accuracy (ACC) of 0.82, significantly outperforming the SVM model (AUC 0.73–0.76) and XGBoost model (AUC 0.78–0.80) in the same study area. Meanwhile, RF can directly output the contribution rate of each evaluation factor and identify core driving factors such as watershed area and mean annual precipitation, whereas SVM and XGBoost can hardly quantify the synergistic mechanism of multiple factors, resulting in weak interpretability. Furthermore, RF has stronger robustness to noisy data and imbalanced samples. Under the imbalanced condition in this study (33 watersheds with debris flows versus 628 watersheds without debris flows), RF still maintains stable assessment results, avoiding the bias amplification prone to XGBoost and the distortion of classification boundaries in SVM.

In summary, the RF model is more suitable for debris flow susceptibility assessment at the watershed unit scale in the upper reaches of the Fujiang River. It outperforms XGBoost and SVM in accuracy, robustness and mechanism interpretation, and can serve as a preferred model reference for watershed-scale geological hazard susceptibility assessment in alpine and canyon regions of southwest China.

### Future research directions and prospects

Altogether, future research can be deepened in the following aspects. First and foremost, it is advisable to integrate high-resolution Sentinel-2 imagery and unmanned aerial vehicle (UAV) survey data to unify factor resolution and enhance evaluation accuracy in local small watersheds. Apart from that, it is preferable to introduce dynamic factors such as seismic ground motion parameters and land use changes, and construct a dynamic evaluation system combined with time-series NDVI to quantify the coupled effects of human activities and natural changes. In line with emerging trends in Applied Sciences special issues on "Intelligent Technologies in Geotechnical Engineering and Geological Hazards" and "Applications of Artificial Intelligence in Geotechnics and Engineering Geology," future work should also explore the integration of physics-informed AI-driven methods that combine machine learning with hydro-mechanical coupling models^41,46,47^. Such approaches can better capture the complex fracture–seepage interactions and permeability evolution processes that govern debris flow initiation under rainfall and seismic triggers^48^. Moreover, it holds pivotal significance to compare the performance of the RF model with other models, such as XGBoost and Support Vector Machine (SVM) to screen the optimal model for mountainous basins. Last but not least, grounded in the evaluation results, delineate differentiated prevention and control zones and propose comprehensive prevention and control schemes that combine engineering governance with non-engineering measures, considering terrain and socioeconomic characteristics. This will provide more practical technical support for the construction of ecological barriers and land space planning in the upper reaches of the Yangtze River.

In addition, field investigations will be conducted targeting different susceptibility zones in the upper reaches of the Fujiang River. Targeted risk prevention and control strategies will be proposed in line with on-site conditions, so as to link the research results with engineering practice and provide actionable support for regional disaster mitigation.

## Data Availability

The data used in this study are derived from the following public sources: (1) In addition to the mudslide disaster sites in the upper reaches of the Weijiang River, which are obtained by the Sichuan Provincial Department of Natural Resources, the applicability of these data is limited. They involve coordinates and are authorized by this study, so the disaster point data is not disclosed. (2) Data source of China’s administrative divisions: https://cloudcenter.tianditu.gov.cn/administrativeDivision (this data cannot be linked due to the restrictions of the external network, but the author confirms that the data set used during the current research is obtained and used at reasonable request). (3) DEM data set source: https://www.gebco.net/data_and_products/gridded_bathymetry_data/. (4) Source of river water system data: https://zenodo.org/records/13841910. (5) Source of temperature data: https://www.ncei.noaa.gov/data/global-summary-of-the-day/archive/. (6) Precipitation data: https://www.ncei.noaa.gov/data/global-summary-of-the-day/archive/. (7) NDVI data set: 10.11888/Terre.tpdc.300328. (8) Land use change data: Professors Yang Jie and Huang Xin from Wuhan University published (10.5281/zenodo.18180184). Further details can be found in the original publications.
